# Comparative metabolomics provides novel insights into correlation between dominant habitat factors and constituents of *Stellaria Radix* (*Stellaria dichotoma* L. var. *lanceolata* Bge.)

**DOI:** 10.3389/fpls.2022.1035712

**Published:** 2022-11-25

**Authors:** Zhenkai Li, Hong Wang, Lu Feng, Le Song, Yongping Lu, Hongying Li, Yanqing Li, Gege Tian, Yan Yang, Haishan Li, Xiangui Mei, Li Peng

**Affiliations:** ^1^ School of Life Sciences, Ningxia University, Yinchuan, China; ^2^ State Key Laboratory of Crop Biology, College of Agronomy, Shandong Agricultural University, Taian, Shandong, China; ^3^ Ningxia Institute of Meteorological Sciences, Yinchuan, China

**Keywords:** *Stellaria dichotoma* L. var. *lanceolata* Bge., Metabolomics, Origin, Habitat factors, Genuineness, Co-expression network analysis

## Abstract

*Stellaria dichotoma* L. var. *lanceolata* Bge. (SDL) is the original plant of the traditional Chinese medicine Yinchaihu (*Stellaria Radix*). It is mainly distributed in the arid desert areas of northwest China, which is the genuine medicinal material and characteristic cultivated crop in Ningxia. This study aims to analyze the effects of different origins on SDL metabolites and quality, as well as to screen the dominant habitat factors affecting SDL in different origins. In this study, metabolites of SDL from nine different production areas were analyzed by ultra-high performance liquid chromatography-quadrupole time-of-flight mass spectrometry (UHPLC-Q-TOF MS) based metabolomics. And field investigations were conducted to record thirteen habitat-related indicators. Results showed that 1586 metabolites were identified in different origins, which were classified as thirteen categories including lipids, organic acids and organic heterocyclic compounds derivatives. Multivariate statistical analysis showed that the metabonomic spectra of SDL from different origins had various characteristics. What’s more, co-expression network correlation analysis revealed that three metabolites modules (MEturquoise, MEbrown and MEblue) were more closely with the habitat factors and 104 hub metabolites were further screened out as the habitat-induced metabolite indicators. Besides, soil texture, soil pH value and soil total salt content were found as the dominant habitat factors which affect SDL metabolites. In conclusion, the study showed different habitat factors had various effects on SDL’s quality and established relationship between them, which provide reference for revealing SDL’s genuineness formation mechanism and guiding industrial crops practical production by habitat factors selection.

## 1 Introduction

Yinchaihu (*Stellaria Radix*) is a kind of Chinese herbal medicine, which is used to clear deficient heat and infantile malnutrition with fever (Li et al., 2020; Chinese Pharmacopoeia Commission, 2020). In modern medicine, it has been found to have good medical prospects such as anti-inflammatory, anti-allergic and anti-cancer and to be rich in active ingredients such as sterols and flavonoids (Ba et al., 2018; Zhang et al., 2019a; Zhang et al., 2019b; Li et al., 2020; Dong et al., 2021). *Stellaria dichotoma* L. var. *lanceolata* Bge.(SDL) is the original plant of Yinchaihu, and its dry roots are raw materials of Yinchaihu (Chinese Pharmacopoeia Commission, 2020). SDL is mainly distributed in semi-arid and arid areas in China and is concentrated in Ningxia, Inner Mongolia, Shanxi. Over the past few decades, with the scarcity of SDL wild resources, people began to explore cultivation and production methods of SDL. Ningxia took the lead in domesticating SDL successfully in the 1980s. Since then, SDL has gradually been developed into a medicinal crop and developed into a genuine medicinal material of Ningxia. Besides, SDL is extended to areas with harsh environments such as central arid areas due to its excellent drought and barrenness tolerance. At present, the largest SDL planting base in China has been built in Tongxin County, Ningxia. The cultivation of SDL has eased the shortage of resources of wild medicinal herbs, brought economic, social and ecological benefits to the cultivation sites and become an important source of economic income for local farmers.

The genuine medicinal materials have been recognized as high-quality Chinese herbs with excellent efficacy which were produced in a specific region since ancient times (Yuan and Huang, 2020). The genuineness is the unique attribute of genuine medicinal herbs, and the habitat is an important manifestation of the genuineness of medicinal herbs and an important factor for the formation of their quality. The secondary metabolites of medicinal plants are the material basis for the therapeutic effects of Chinese herbal medicines. And, different habitat factors affect the quality and therapeutic effects of medicinal herbs by regulating the formation and accumulation of secondary metabolites in medicinal plants (Mudge et al., 2016; Jiang et al., 2020), which exerts influence on medicinal herbs’ genuineness. For SDL, changes in the production methods have also led to the migration and change of its origins. In addition to the central arid area, SDL has also started to be cultivated and produced in the non-arid areas south of the central arid area in Ningxia. However, it has not been scientifically verified whether the migration of origin and the change of habitat have impacts on the the secondary metabolites of SDL and the quality of the medicinal herbs.

Metabolomics is a technique for qualitative and quantitative analysis of all metabolites in living organisms, and has been widely used in research fields such as quality evaluation of traditional Chinese medicine, formation mechanism of genuineness, screening biomarkers and new drug development due to its advantages with high-throughput and high sensitivity (Nicholson and Wilson, 2003; Nicholson and Lindon, 2008). Besides, non-targeted metabolomics analysis is an important mode which is based on a high-resolution mass spectrometer and is capable of an unbiased, large-scale and systematic detection for various metabolites in samples, reflecting the changes of metabolic levels in organisms to the greatest extent (Christ et al., 2018).

Co-expression network analysis (CNA) is a method of systems biology for analyzing the correlation of gene, protein or metabolite expression in multiple samples, which can classify a large amount of biological information into different information modules and conduct a correlation analysis with phenotypes. The method takes full advantage of the overall omics information, but also converts a large amount of biological information into a module-phenotype association, eliminating the need for multiple hypothesis testing correction (Langfelder and Horvath, 2008; Qing et al., 2020). CNA analysis was first applied to genomics analysis. Later, with the effective practice of Matthew and others in correlating tomato metabolomics data with genetic background analysis (DiLeo et al., 2011), this method has begun to be widely used in metabolomics analysis and become a powerful means of helping metabolomics data explain more scientific problems.

In this study, SDL samples were collected from nine origins with typical habitat characteristics. And their metabolites differences were identified by ultra-high performance liquid chromatography-tandem time-of-flight mass spectrometry (UHPLC-Q-TOF MS) based metabonomics. Moreover, multivariate statistical analysis and CNA analysis were adopted to analyze the correlation between the metabolites and environmental habitats factors, so as to explore the impact of different origins on SDL metabolites and screen the main habitat factors.

## 2 Materials and methods

### 2.1 SDL collection and processing

**Figure 1:**
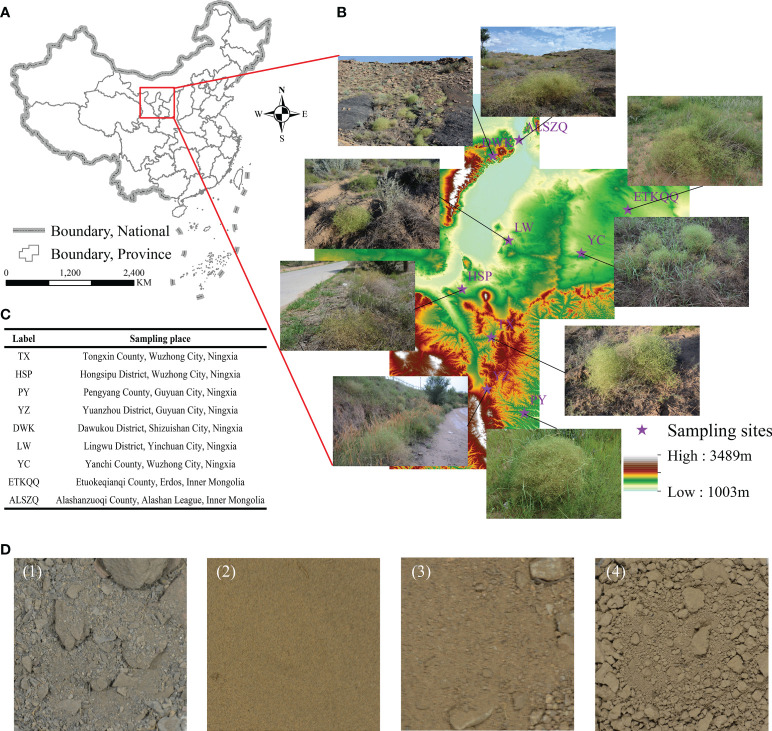
Specific information of different SDL sampling sites. (**A–C** are the information and pictures of the sampling sites. **(D)** is the soil of different sampling sites, of which (1) is gravel soil, from DWK; (2) sandy soil, from LW, YC, EKTQQ and ALSZQ; (3) loam soil, from HSP; (4) clayey soil, from TX, PY and YZ.).

The materials were collected from nine different producing areas (**Figure 1**) in August 2020 and were all identified as roots of *Stellaria dichotoma* L. var. *Lanceolata* Bge in this study. The specific sampling location information is shown in **Figure 1** Random sampling shall be conducted at each sampling point for six repetitions. The roots of SDL must be naturally dried to constant weight, crushed and sieved through 40 meshes before storing them in a dark and refrigerated place for later use. The TX sample, growing for three years in a test site, had no field cultivation such as fertilization, water after it was sowed, the PY, YZ and HSP samples were collected from farmland that had been abandoned for many years and the other samples were from natural habitats.

### 2.2 Investigation on habitat factors in different origins

#### 2.2.1 Investigation on soil physical and chemical properties

The soil samples were collected around the SDL. Soil samples were collected by establishing a 30 × 30 cm sample square with SDL as the center, each sample square was sampled with a shovel at a depth of 500 g from 0-20 cm, 20-40 cm and 40-60 cm respectively, and the soil from the three depths was mixed to form one soil sample. Three biological replicates of soil samples were taken from each origin. These soil samples were naturally dried and stored in a refrigerated area away from light for the subsequent testing. Soil particle size was determined by soil sieve and laser particle size analyzer, respectively. The type of soil texture was classified by referring to the *National Standard of the People’s Republic of China* “*Engineering Classification Standard for Soil”(GB/T50145-2007)*. The total salt content of soil suspensions was determined by means of the conductivity method from “*Soil Testing Part 16: Determination of Total Water Soluble Salts in Soil*” (NY/T1121.16-2006). The pH value of soil suspensions was determined by using the pH meter method from “*Soil Testing Part 2: Determination of Soil pH*” (NY/T 1121.2-2006). And, the organic matter content of the soil was determined by referring to “*Soil Testing Part 6: Determination of Soil Organic Matter*” (NY/T1121.6-2006).

#### 2.2.2 Meteorological factors collection

Meteorological factors were collected from meteorological stations closest to the collection sites, corresponding to station numbers 53811, Y3417, Y2810, Y3028, Y1710, Y1351, Y2534, 53730 and 53602, respectively. The data mainly includes the annual average precipitation, the annual average temperature, the highest temperature in July, the average temperature in July, the lowest temperature in January and the average temperature in January.

### 2.3 Determination of chemical composition of SDL

#### 2.3.1 Determination of the extract content

The cold leaching method with methanol was used to determine the extract content of SDL as the general rule “2201 *Determination of extract*” in “*Pharmacopoeia of the People’s Republic of China (2020 edition)*”. The specific method is: the methanol solvent is used to extract the SDL sample, the methanol in the resulting extract is evaporated, the methanol extract is obtained, and the extract content of the sample is calculated by weighing method.

#### 2.3.2 Determination of total flavonoids content

The determination of total flavonoids content was based on the previous reported methods (Guo H. et al., 2020) and improved accordingly. The specific process was as follows: 2.00 g of the medicinal powder sample was weighed into a centrifuge tube, 25 ml of 95% ethanol was added and the supernatant was extracted by ultrasonication for 30 min and then, the supernatant was separated. The residue was then added with 25 ml of 95% ethanol to continue ultrasonic extraction for 15 min, and the supernatants of the two extractions were mixed as the test sample solution. The total flavonoids content of the herb was determined by measuring the absorbance value of the sample at 496 nm by using rutin as the control.

#### 2.3.3 Determination of total sterols content

The determination of total sterols content was based on the previous reported methods (Zhang et al., 2012) and improved accordingly. The specific process was as follows: 0.50 g medicinal powder sample was weighted into a 25 ml volumetric flask and then to add 20 ml of chloroform, which was extracted by ultrasonication for 20 min. After 20 min of the ultrasonic extraction, supernatant was acquired. The second step was to dilute the supernatant with chloroform to the scale before shaking it well and filtering it, thus obtaining the test sample solution. The final step was to determine the total sterols content of the herb by measuring the absorbance value at the wavelength of 546 nm by using *α*- spinasterol as the control.

#### 2.3.4 Metabolite detection

The metabolomic analysis of the herbs was carried out by Shanghai Applied Protein Technology Co., Ltd. The method was as follows: the first step was to grind herbal powder in liquid nitrogen, after which 200 mg of the powder was weighted into a 2 ml centrifuge tube. Following this, 70% methanol aqueous extraction solution was added to the centrifuge tube and then vortexed it thoroughly. The second step was to extract the solution, before drying extraction solution under vacuum to get extract. Then, the extract must be stored at -80°C. The next step was to dissolve the extract with 40% acetonitrile water solution to get supernatant and then to analyze the metabolic compositions in the supernatant. The separation was performed with an Agilent 1290 Infinity LC HILIC column; column temperature 25°C; flow rate 0.5 ml/min; injection volume 2 μl; mobile phase composition A: water + 25 mM ammonium acetate + 25 mM ammonia, B: acetonitrile; gradient elution procedure as follows: 0~0.5 min, 95% B The gradient elution procedure was as follows: 0 ~ 0.5 min, 95% B; 0.5~7 min, B linearly varied from 95% to 65%; 7~8 min, B linearly varied from 65% to 40%; 8~9 min, B maintained at 40%; 9~9.1 min, B linearly varied from 40% to 95%; 9.1~12 min, B maintained at 95%. During the whole process of analysis, the sample was placed in an autosampler at 4°C. And, mass spectrometric analysis was performed with triple TOF 6600 mass spectrometer, and the positive (pos) and negative (neg) ion modes of electrospray spray ionization (ESI) were used for detection. The metabolites were identified by matching the retention time, molecular weight (error <25 ppm), secondary fragmentation spectrum, collision spectra and other information of metabolites by means of searching a local self-built standards database established by Shanghai Applied Protein Technology.

### 2.4 Data analysis

Multivariate statistical analyses such as hierarchical cluster analysis (HCA), principal component analysis (PCA), and K-means cluster analysis were performed by R software (www.r-project.org/). The significant changed metabolites(SCM) were screened by Fold Change Analysis(*FC* analysis), *T* test and Orthogonal Partial Least Squares Discriminant Analysis(OPLS-DA) The specific screening criteria were *VIP* > 1, *FC* > 1.5 or *FC* < 0.67 and *p* < 0.05. The KEGG (Kyoto Encyclopedia of Genes and Genomes, https://www.kegg.jp/) database was used for annotation and functional enrichment analysis of SCMs. Based on the CNA method, metabolite co-expression networks and modules were constructed. Based on the criteria of the correlation coefficient R value being closer to ± 1 and the correlation test *p* being less than 0.05, correlation analysis was performed for co-expression modules and thirteen major habitat factors. The expression of metabolites in the screened key modules in all samples was subjected to cluster heat map analysis to compare the distribution of eigenvalues of each module in all samples. The hub metabolites were further screened by analyzing the correlation(*r*) between metabolites in key modules and modules, and the correlation between metabolites in key modules and traits.

## 3 Results and analysis

### 3.1 Habitat characteristics of SDL in different origins

#### 3.1.1 Spatial distribution analysis

As shown in **Figure 1** and **Table 1**, the nine SDL origins were distributed at 35.00^°^N-40.00^°^N, 105.00^°^E-109.00^°^E, altitude 1 050.00-1 650.00 m. The naturally distributed samples grew at 38.00^°^N above and below 1 300 m above sea level; samples from farmland or abandoned farmland grew at 38.00^°^N below and above 1 300 m above sea level.

**Table 1:** Habitat analysis of SDL from different origins.

Label	Latitude, N	Longitude, E	Elevation, m	average annual precipitation, mm	average annual temperature, °C	highest temperature in July, °C	average temperature in July, °C	lowest temperature in January, °C	average temperature in January, °C	Soil texture	Total salt content, g/kg	pH value	Organic matter content,g/kg
TX	36.76^°^	106.36^°^	1558	311.15 ± 65.95b	8.78 ± 1.08c	29.29 ± 1.35cd	22.31 ± 0.73de	-15.33 ± 1.16b	-8.52 ± 1.55c	clayey soil	3.31 ± 0.14 bc	8.71 ± 0.11 cd	7.00 ± 0.85 b
HSP	37.40^°^	105.97^°^	1335	148.72 ± 64.87c	10.81 ± 2.72ab	32.62 ± 1.09b	24.84 ± 1.41ab	-11.29 ± 0.99a	-7.09 ± 4.00abc	loam soil	5.72 ± 0.37 bc	8.49 ± 0.15 de	1.01 ± 0.08 d
PY	35.75^°^	106.80^°^	1395	583.17 ± 145.53a	8.90 ± 0.47bc	29.10 ± 2.22cd	21.43 ± 1.25e	-10.63 ± 1.63a	-4.82 ± 1.58a	clayey soil	4.61 ± 0.32 bc	8.51 ± 0.05 de	8.03 ± 0.94 ab
YZ	36.07^°^	106.30^°^	1624	475.47 ± 182.34a	9.11 ± 1.23bc	27.67 ± 2.00d	21.37 ± 0.67e	-10.42 ± 1.65a	-5.03± 1.76ab	clayey soil	6.13 ± 0.51 bc	8.62 ± 0.09 cde	3.16 ± 0.37 c
DWK	39.18^°^	106.38^°^	1300	166.73 ± 56.52bc	11.07 ± 1.44a	32.48 ± 0.65b	25.80 ± 1.01a	-11.96 ± 0.79a	-6.34 ± 1.28abc	gravelly soil	29.77 ± 4.22 a	7.29 ± 0.47 e	8.29 ± 1.34 a
LW	38.05^°^	106.59^°^	1273	304.40 ± 212.85bc	9.22 ± 1.12abc	30.80 ± 0.57bc	23.51 ± 0.56cd	-11.76 ± 0.75a	-6.12 ± 1.10abc	sandy soil	2.43 ± 0.35 c	9.17 ± 0.03 a	0.72 ± 0.05 d
YC	37.88^°^	107.56^°^	1298	190.25 ± 73.14bc	8.46 ± 1.61c	31.50 ± 1.42b	24.01 ± 0.40bc	-14.59 ± 1.27b	-7.83 ± 1.72bc	sandy soil	2.90 ± 0.21 c	8.92 ± 0.07 bc	0.65 ± 0.01 d
ETKQQ	38.46^°^	108.18^°^	1290	266.10 ± 43.13bc	8.97 ± 0.18bc	36.48 ± 1.62a	24.07 ± 0.58bc	-23.18 ± 1.28d	-8.05 ± 1.62c	sandy soil	7.82 ± 0.17 b	8.67 ± 0.11 cd	1.08 ± 0.06 d
ALSZQ	39.39^°^	106.73^°^	1069	248.02 ± 47.00bc	9.50 ± 0.21abc	35.57 ± 1.56a	24.08 ± 0.50bc	-17.73 ± 3.37c	-7.00 ± 2.01abc	sandy soil	2.51 ± 0.08 c	8.31 ± 0.06 d	1.07 ± 0.08 d

The lowercase letters after the data indicate significant differences in habitat factors between different SDL origins at p<0.05 levels.

#### 3.1.2 Meteorological factor analysis

The average values of the six meteorological factors collected from 2015 to 2020 are shown in **Table 1**. The annual precipitation for the nine producing areas ranged from 183 cm to 583 cm. The average annual temperature ranged from 8.5°C to 11.1°C, the maximum temperature in July ranged from 27.7°C to 36.5°C and the average temperature in July ranged from 21.4°C to 25.8°C. The minimum temperature in January ranged from -23.2°C to -10.4°C and the average temperature in January ranged from -8.5°C to -4.8°C. Besides, the significance analysis showed that the six meteorological factors showed different levels of variation due to different producing areas.

#### 3.1.3 Analysis of the physical and chemical properties of soils

As shown in **Figure 1D**, the soil from DWK (label refers to origin, the same below) habitat showed a dark grey color and the rest were yellowish brown. The soils from the DWK, ETKQQ, YC, LW, ALSZQ and HSP habitats were relatively loose, while soils from TX, PY and YZ were highly viscous and have soil agglomeration. The physical and chemical properties of soils were further analyzed and the results are shown in **Table 1**. In terms of soil texture, total salt content, pH value and organic matter content, the habitat soils of SDL from different origins were different in varying degrees. The results of the soil texture analysis showed that DWK had the largest proportion of large grained soils, which was classified as gravelly soils. Then, soils from ETKQQ, YC, LW and ALSZQ were classified as sandy soils. TX, PY and YZ had a higher proportion of finer grained soils, which was classified as clayey soil. The soil texture of HSP is between sandy and clayey soil, and is classified as loam soil. The total salt content of DWK was 29.77 g/kg which was significantly higher than other producing areas, while the pH value of DWK was 7.29 which was significantly lower than that of others. And, for other producing areas, the total salt content was less than 8.00 g/kg and pH values ranged from 8.31 to 9.17. In addition, the organic matter content of DWK, PY and TX was significantly higher than that of other origins, which were 8.29 g/kg, 8.03 g/kg and 7.00 g/kg respectively, followed by YZ with 3.16 g/kg, and the rest of the samples were around 1.00 g/kg.

### 3.2 Analysis of the characteristics of medicinal materials, methanol extract, total sterols and total flavonoids content of SDL from different origins

#### 3.2.1 Characteristics of medicinal materials

**Figure 2:**
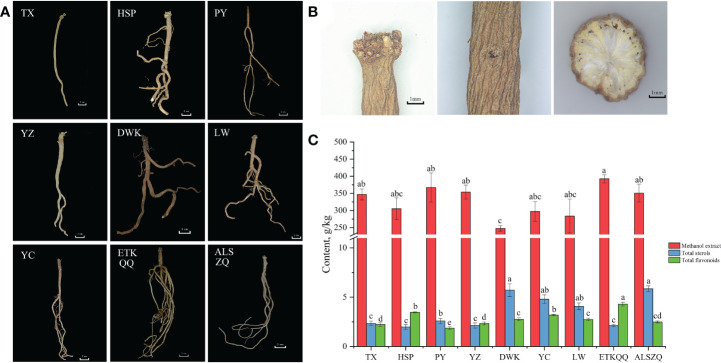
The characteristics of medicinal materials, methanol extract, total sterols and total flavonoids content of SDL in different origins. (**A** showed the overall morphology of SDL from different origins. **B** showed the main medicinal characteristics of SDL, including ‘shayan’, ‘zhenzhupan’ and yellow and white cross-section of the radial texture from left to right. **C** is the content of methanol extract, total sterols and total flavonoids of SDL from different origins,and the lowercase letters in the bar chart indicate significant differences in the content of components of SDL between different origins at the *p*< 0.05 level. The same letter indicates no significant difference, while different letters indicate significant difference.).

The “shayan” (hole-like or disk-like depression), “zhenzhupan” (wart-like protruding buds, stems or rhizome stumps) and yellow and white cross-section of the radial texture are the most important characters of SDL. As shown in **Figures 2A, B**, the SDL from the nine origins in this study all had “shayan”, “zhenzhupan” and yellow and white cross-section of the radial texture. The difference is that SDL collected from wild natural habitats was darker in colour (DWK, LW, YC, ETKQQ and ALSZQ) and was light brown or brown. SDL collected from test or abandoned agricultural fields were lighter in colour (TX, HSP, PY and YZ) and were pale yellow or light brownish-yellow. In addition, SDL collected from clayey soil (TX, PY and YZ) were mostly long-columnar in shape and had no or few branches. SDL collected from loam, sandy or gravelly soils (HSP, LW, YC, ETKQQ, ALSZQ and DWK) were more variable in character and generally had multiple branches. In summary, SDL from different origins has the basic characteristics of medicinal herbs, but different habitats may affect the overall color and morphology of SDL.

#### 3.2.2 Methanol extract, total sterols and total flavonoids content

The methanol extract is the content determination index for evaluating SDL as stipulated in the *Chinese Pharmacopoeia*, and total sterols and total flavonoids content are the most commonly used indexes for evaluating the quality of SDL at present (Li et al., 2020). In this study, the three indexes of SDL from different origins were tested, and the determination results are shown in **Figure 2C**. The content of SDL methanol extract, from different origins reached the 20% index stipulated in the *Chinese pharmacopoeia*. And, the lowest content of DWK was 24.77% and the highest content of ETKQQ was 39.27%, while others did not show significant differences (P < 0.05). The total sterol content was the highest in ALSZQ and DWK with 5.85 g/kg and 5.71 g/kg respectively, while the total sterols content of HSP, ETKQQ, YZ and TX were significantly lower than the other samples. The total flavonoids content of ETKQQ was 4.29 g/kg which was significantly higher than the samples from other origins. The sample with the lowest total flavonoid content was PY, which was 1.93 g/kg and significantly lower than samples from other origins. In summary, in addition to the differences in the methanol extracts of SDL from different habitats, there were greater differences in the contents of total flavonoids and total sterols. It is speculated that there may be more different substances in SDL from different origins.

### 3.3 SDL metabolites and quality control

**Figure 3:**
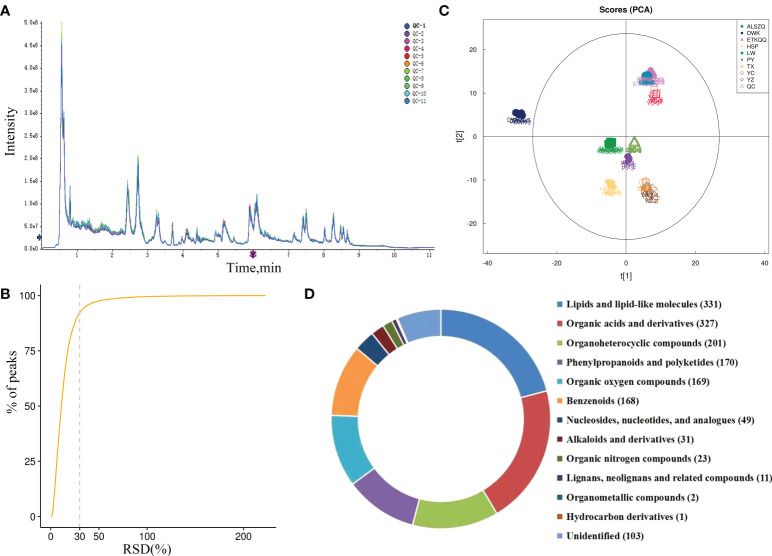
Quality control and identification analysis of metabolite detection. (**A** is TIC diagram in pos ion mode; **B** is characteristic peak variation coefficient in pos ion mode; **C** is the PCA of metabolites detected in pos ion mode; **D** is the category and pie chart of identified metabolites.).

The total ion chromatograms (TIC) of all QC samples were compared by overlapping spectra, as shown in **Figure 3A** and **Supplementary Figure 1A**, and the response intensities and retention times of the peaks basically overlapped, indicating good instrument precision throughout the experiment. The proportion of QC samples with relative standard deviation (RSD) less than 30% of the characteristic peaks exceeded 70% (**Figure 3B** and **Supplementary Figure 1B**), indicating a good stability of the instrument. PCA analysis was performed on all samples and QC samples. As shown in **Figure 3C** and **Supplementary Figure 1C**, the QC samples were closely clustered together, indicating that the samples had good repeatability. In addition, the results of PCA analysis also showed that DWK had significantly different principal component characteristics. ETKQQ, LW and YC had similar principal component characteristics. And TX, YZ, PY, ALSZQ and HSP had closer principal component characteristics.

The metabolites were identified by searching the local self-built standards database. A total of 1586 substances (**Supplementary Table 1**) were identified from nine origins of SDL and divided into thirteen categories. Among them, 880 substances were identified in pos mode and 706 substances in neg mode. And the categories mainly include lipids and lipid-like molecules (331 species), organic acids and derivatives (327 species), organicheterocyclic compounds (201 species), phenylpropane and polyketone compounds (170 species), organic oxygen compounds (169 species), benzenes (168 species), etc. (**Figure 3D**).

### 3.4 Analysis of SCMs in SDL of different origins

**Figure 4:**
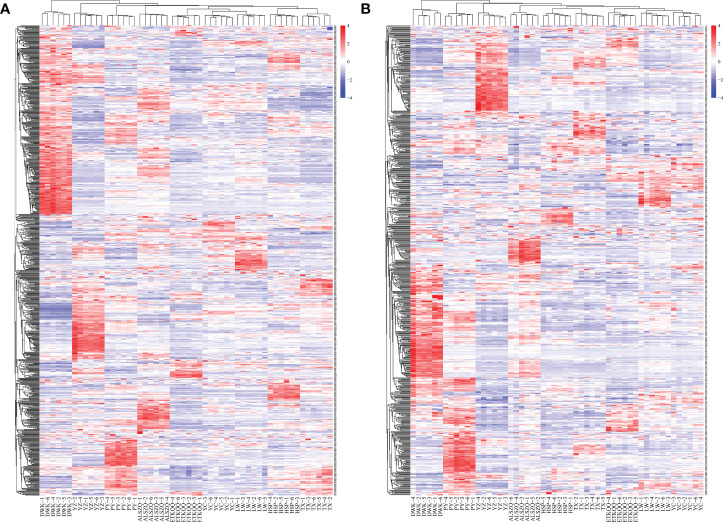
Heatmap of hierarchical clustering of SDL metabolites in habitats of different origin. (**A** is the metabolite detected in pos mode and **B** is the metabolite detected in neg mode).

**Figure 5:**
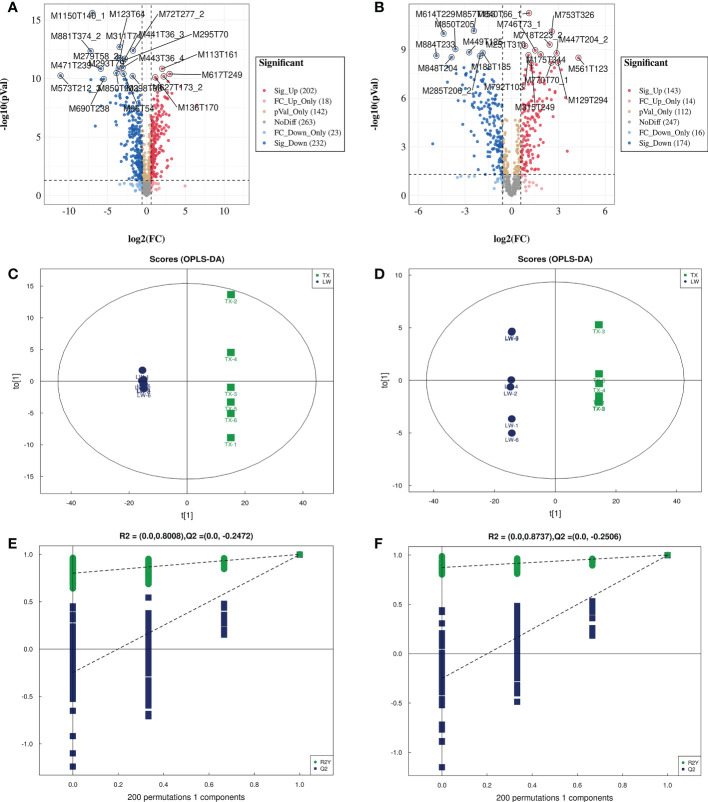
Volcano plot, OPLS-DA and permutation test analysis of differential metabolites of TXvsLW. (**A, C, E** are volcano plot, OPLS-DA and permutation test analysis for detecting metabolites in pos mode, respectively; **B, D, F** are volcano plot, OPLS-DA and permutation test analysis for detecting metabolites in neg mode, respectively.).

**Figure 6:**
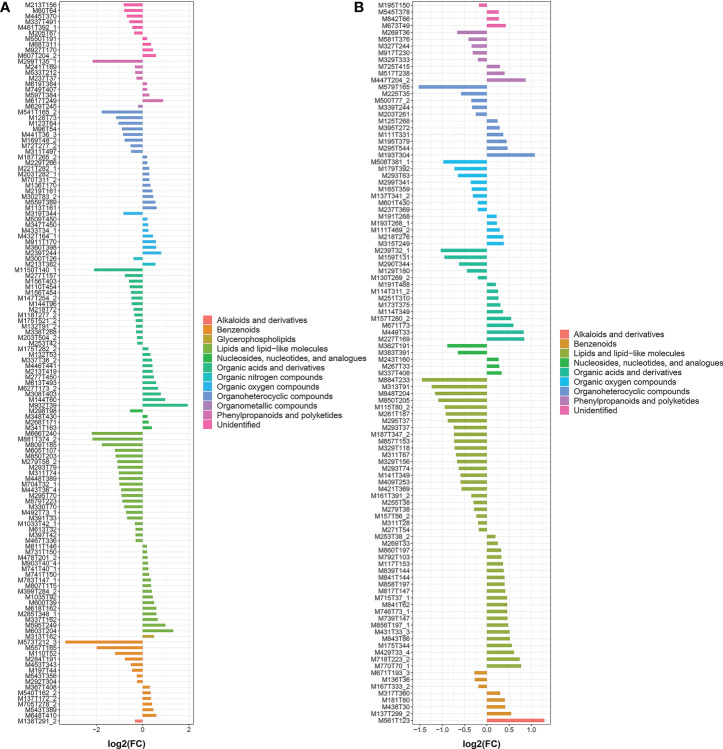
*FC* analysis histogram of SCMs in TXvsLW. (**A** is the metabolite detected in pos mode and **B** is the metabolite detected in neg mode.).

**Figure 7:**
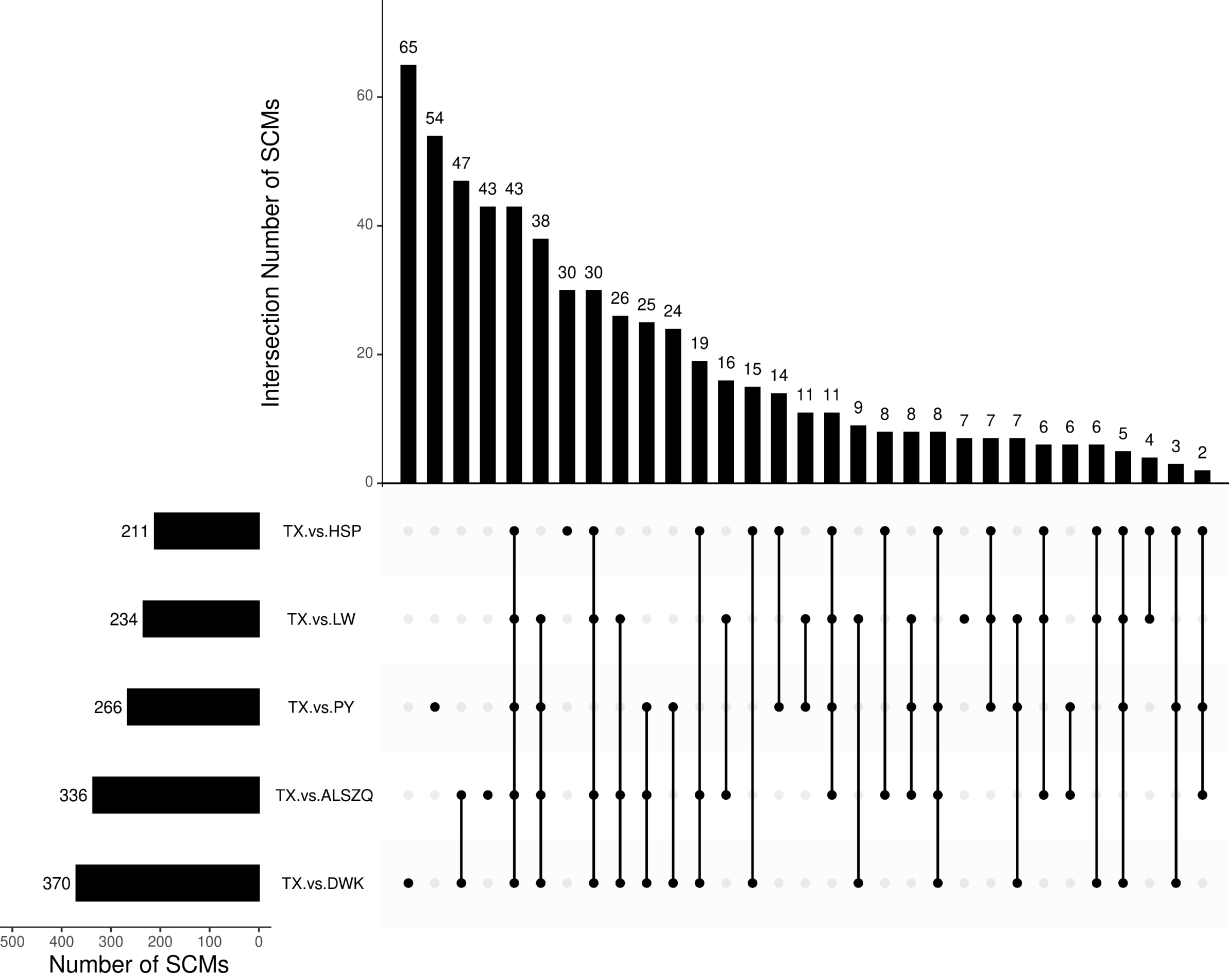
Venn diagram analysis of SCMs in Different Comparison Groups.

**Figure 8:**
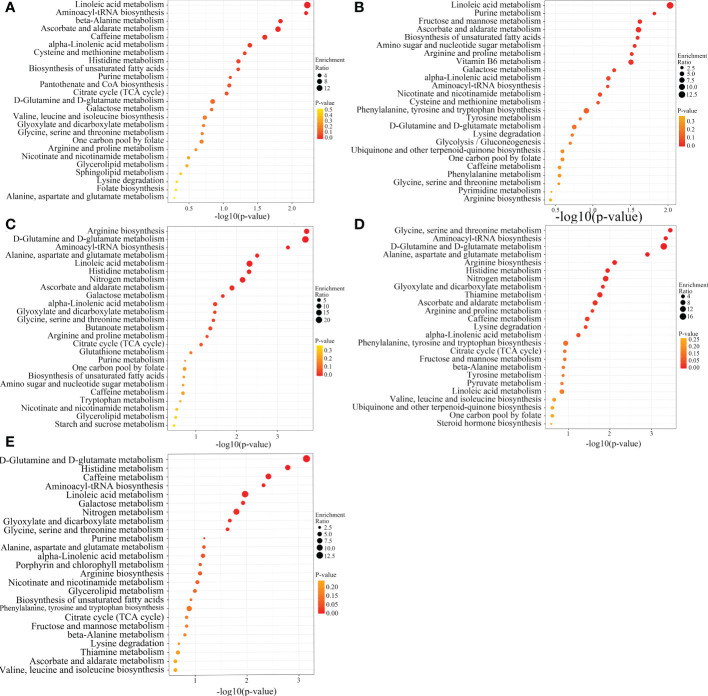
KEGG enrichment analysis of SCMs from different SDL comparison groups. (**A–E** are the ones of TX vs LW, TX vs PY, TX vs HSP, TX vs DWK, and TX vs ALSZQ, respectively.).

A systematic clustering heat map analysis of the ionic intensities of each metabolite was performed for SDL samples of all origins, as shown in **Figure 4**. Result showed that DWK clustered separately with samples from other origins, LW, YC and ETKQQ clustered in a group, and TX and HSP clustered in a group, which was generally consistent with the characteristics showed by the PCA analysis (**Figure 2C** and**Supplementary Figure 1C**). The total ion heat map of metabolites showed significant expression differences for SDL metabolites of different origins. According to the results of principal component analysis and clustering, and habitat characteristics, TX was selected for pairwise comparison with LW, PY, HSP, ALSZQ and DWK samples. The SCMs between TX and other samples were screened by *FC* analysis, *T* test and OPLS-DA, (**Figure 5**, **Supplementary Figures 2**–**4**). The results showed that there were significant differences in SCMs between different comparison groups. The number of SCMs in different comparison groups from high to low is: TX vs DWK (370 species), TX vs ALSZQ (336 species), TX vs PY (266species), TX vs LW (234 species) and TX vs HSP(211 species)(**Figure 6** and **Supplementary Figure 5**). These SCMs were reflected in a variety of classifications such as lipid and lipid-like molecules, organic acids and derivatives, phenylpropanoids and polyketides, etc. Further Venn diagram analysis of all SCMs showed that a total of 43 common SCMs were identified in the five comparison groups (**Figure 7**). In addition, TX vs DWK has 65 unique SCMs; followed by TX vs PY and TX vs ALSZQ, 54 and 43 species, respectively, TX vs LW was the least, only seven species. The KEGG database was further used to annotate and enrich the scm of the five comparison groups. As shown in **Figure 8**, SCMs in different comparison groups are enriched in different signaling pathways, and the significance of the same signaling pathway in different comparison groups is also different. This further indicates that SDLs from different origins have more differences in metabolites and metabolic pathways.

### 3.5 Correlation of SDL metabolites with dominant environmental factors

**Figure 9:**
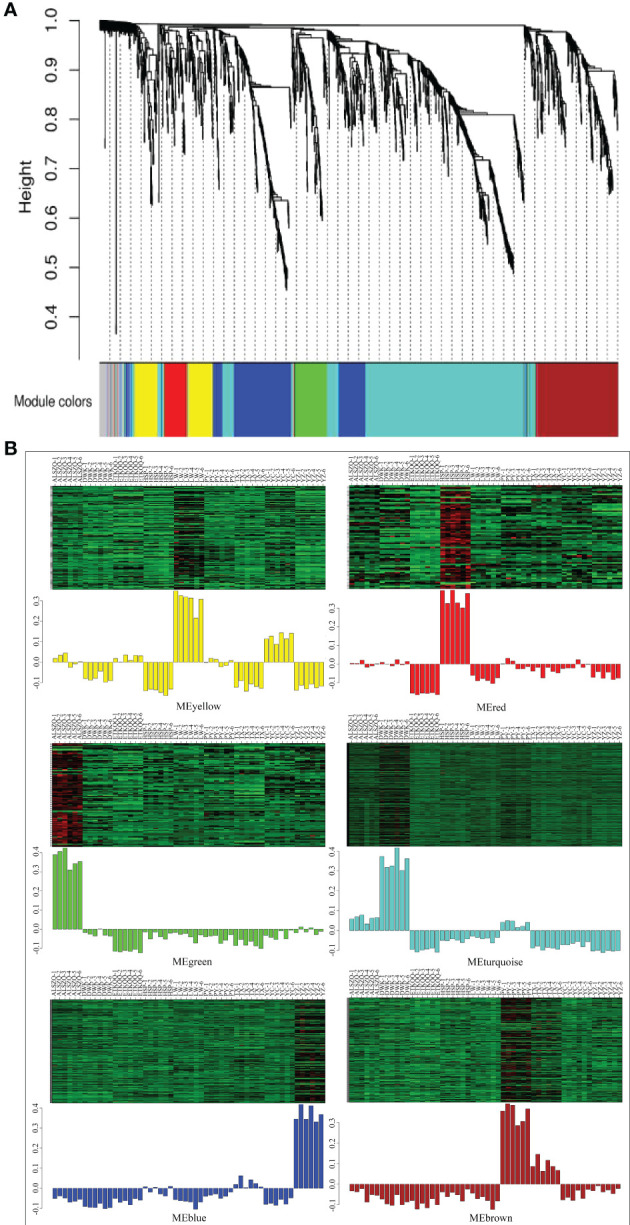
Heat map of metabolite co-expression module division and their correlation with phenotypic traits. (**A** is metabolite co-expression module; **B** is heat map of metabolite expression and the distribution map of module eigenvalues.).

**Figure 10:**
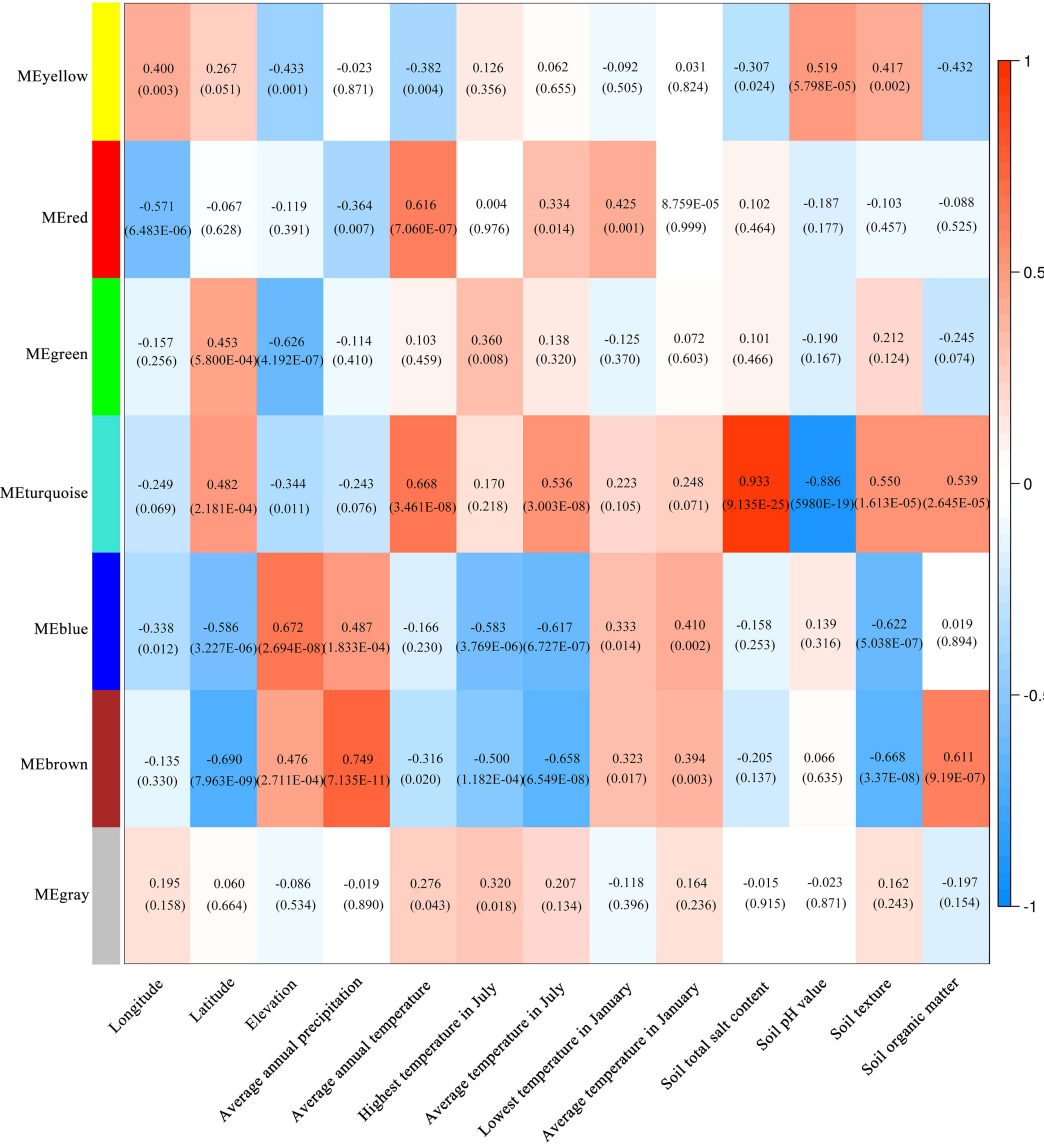
Correlation analysis between module and habitat factors. (The numbers in the colour block in the figure, the top one is the *r*-value and the bottom one (in brackets) is the *P*-value).

The co-expression network of 1586 metabolites were constructed by WGCNA, and the correlation analysis was carried out with a total of thirteen habitat factors, including soil physicochemical properties, meteorological factors, and spatial distribution. As shown in **Figure 9A**, 1586 metabolites were divided into six co-expression modules (MEyellow, MEred, MEgreen, MEturquoise, MEblue and MEbrown) and one module without obvious co-expression relationship (MEgrey). And the heat map of metabolite expression and the distribution map of module eigenvalues (**Figure 9B**) showed that MEyellow from LW and YC had higher up-regulated expression in all samples, MEred from HSP had higher up-regulated expression, MEgreen from ALSZQ had higher upregulated expression, MEturquoise from DWK had higher up-regulated expression, MEblue from YZ had higher up-regulated expression, PY and TX MEbrown had higher up-regulated expression. These specifically expressed metabolite modules could be used as characteristic groups of constituents of SDL in different origins. Further correlation analysis of the seven metabolite modules with the thirteen habitat factors showed (**Figure 10**) that MEturquoise had a significant positive correlation with total salt content of soil(*r*=0.933, *p*<0.05) and average annual temperature(*r*=0.668, *p*<0.05), while a negative correlation with soil pH value(*r*=-0.886, *p*<0.05). MEbrown had a significant positive correlation with average annual precipitation(*r*=0.749, *p*<0.05), and a significant negative correlation with latitude (*r*=-0.690, *p*<0.05)and soil texture(*r*=-0.668, *p*<0.05). MEblue had a significant positive correlation with elevation(*r*=0.672, *p*<0.05). As key modules, these highly correlated modules (MEturquoise MEbrown and MEblue) were metabolite groups that were largely influenced by habitat factors. Significantly, the total salt content, pH value and soil texture, average annual temperature and precipitation, elevation and latitude were the main habitat factors resulting in SDL metabolite differences.

**Figure 11:**
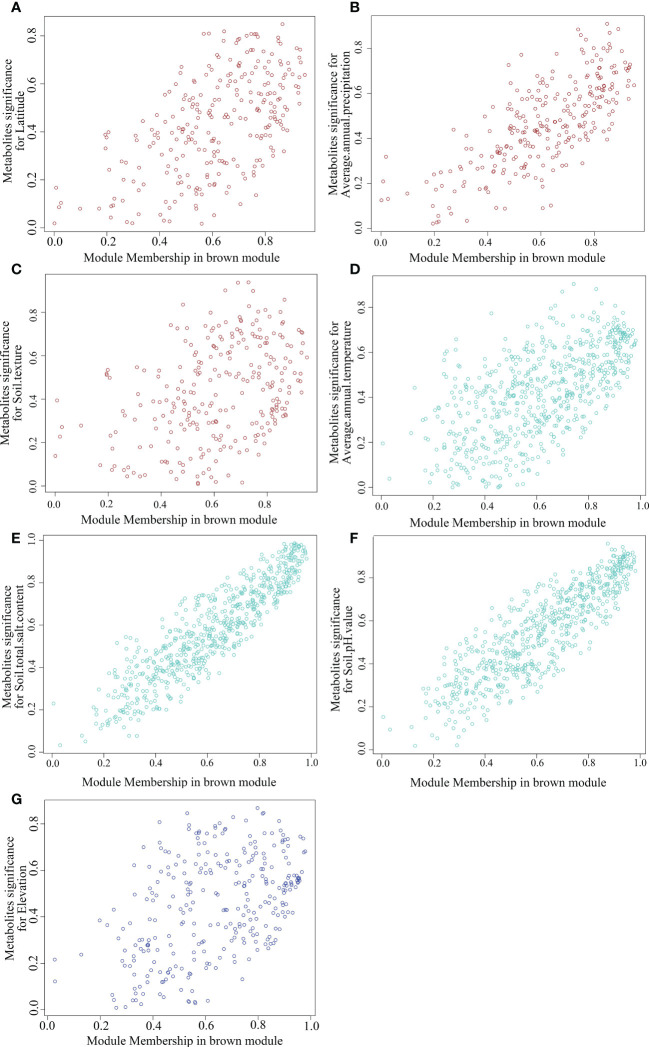
Scatter distribution of module membership vs metabolites significance. (**A–G** are MEbrown-Latitude, MEbrown-Average annual precipitation, MEbrown-Soil texture, MEturquoise-Average annual temperature, MEturquoise-Soil tital salt content, MEturquiose-Soil PH value and MEblue- Elevation, respectively.).

To further screen out the key metabolites with high correlation from the key modules, we analyzed the scatter distribution of metabolite significance (correlation between metabolites and habitat factors) and module membership (correlation between metabolites and modules) in each module (**Figure 11**). And the key metabolite was filtered out based on the metrics of metabolite significance and module membership (Top50 and *p*<0.05). As shown in **Supplementary Table 2**, 104 species hub metabolites were screened, including 22 species lipids and lipid-like molecules, 13 species benzenoids, 17 species organic oxygen compounds, 15 species organic acids and derivatives, 14 species organoheterocyclic compounds and 9 species phenylpropanoids and polyketides, etc. Morever, the largest number of hub metabolites significantly associated with soil texture was 37 species, followed by soil total salt content (34 species), soil pH value (34 species), average annual precipitation (25 species), elevation(14 species), average annual temperature (11 species) and latitude (11 species). The hub metabolites are probably to be the main metabolites of SDL in response to different habitat factors. On the contrary, the corresponding habitat factors may be the dominant factors which affect SDL’s quality.

## 4 Discussion

In this study, habitat surveys of nine origins of SDL revealed that there were significant differences among them especially in the soil physicochemical properties. In terms of medicinal properties, further analysis revealed that the MeOH extract, total sterols and total flavonoids content in SDLs from different origins were also obvious different. Meanwhile, metabolomics analysis showed that SDL from different origins contained different metabolite characteristics and showed rich diversity. Among which the metabolic characteristics of LW, YC and ETKQQ were more similar, and the difference of DWK was the largest. In addition, the results of SCMs screening showed that the metabolites of TX were similar to those of LW and HSP. At the same time, KEGG enrichment analysis also enriched the SCMs of different comparison groups into different signaling pathway. These results further indicate that different origins have significant effects on the production, accumulation and signaling pathways of SDL metabolites. This is consistent with the conclusion of metabolomics analysis of medicinal plants such as *Chrysanthemum* (Zou et al., 2022) and *American ginseng* (Si et al., 2021), from different origins. Furthermore, bioactive substances are the material basis for medicinal plants (Li et al., 2018) to exert their efficacy and are also important indicators for evaluating the quality of medicinal materials. (Li et al., 2018). However, SDL has not yet established systematic quality evaluation system and its bioactive material basis research is lagging behind. Herein, results showed that all of the methanol extract content of SDL from different habitats reached the requirements of *Chinese Pharmacopoeia* (20%), there were still more significant differences in total sterols, total flavonoids and other metabolites. These differential metabolites contain a variety of bioactive ingredients, such as organic acids, phenol propane, polyketide compounds, lipid and their derivatives, which may lead to different efficacy effects and herb quality. Therefore, the in-depth research on SDL quality biomarkers and habitat selection should be paid more attention.

The bioactive substances are the products of long-term adaptation to specific environments for medicinal plants, which are mostly the secondary metabolites accumulated in response to habitat abiotic and biotic stresses (Huang and Guo, 2007; Li et al., 2019). Consequently, the same plant will also metabolize and accumulate various secondary metabolites in different habitats. The SDLs were collected from different ecological regions in this study with complex and diverse habitat differences in the soil environment, climate and spatial distribution. Therefore, the characteristics of differential metabolites in SDL from different habitats may be affected by the interaction of multiple habitat factors. In this study, SDL metabolites were correlated with thirteen habitat indicators by the WGCNA analysis. And, the results showed that several metabolite modules (MEturquoise, MEbrown and MEblue)were closely correlated with several habitat indicators such as total soil salt content, pH value, organic matter content, soil texture, annual precipitation, average annual temperature and altitude.

Further screening of the hub metabolites from the metabolite module revealed that the hub metabolites were most associated with soil texture, soil pH and soil salinity. Soil is the material basis for the growth and development of medicinal plants. The physical and chemical properties of soil are important components of soil environment. SDL is a psammophyte. Most of the wild SDLs collected in this study grow in sandy soil, but non-wild SDLs collected from TX, PY, YZ and HSP grow in clayey soil or loam soil. The change of soil texture will not only change porosity and soil water retention capacity, but also affect the soil available nutrients and soil microorganism, and further affect the plant transpiration, photosynthesis, respiration and other physiological and biochemical effects and the accumulation of secondary metabolites (Du et al., 2019; Haruna and Yahaya, 2021). Therefore, different medicinal plants have preferences for soil texture according to their physiological needs. For example, *Scrophularia ningpoensis* is suitable for growing in limestone heavy loam, and loess with deep soil layer is suitable for the growth of *Astragalus membranaceus*, and *Codonopsis pilosula* and *Rehmannia glutinosa* are suitable for growing on fertile sandy soil (Chen and Tan, 2006; Tian et al., 2013). Therefore, whether SDL is suitable to grow in non-sandy soil needs further study. Salt stress is an important abiotic stress affecting secondary metabolism of medicinal plants (Hemanta, 2017). Zhang found that the yield of SDL herbs and the accumulation of total flavonoids and total saponins reached the maximum when the soil salt content was 0.3% (Zhang et al., 2017). Soil pH is also an important environmental factor affecting the metabolites of medicinal plants (Du et al., 2013), and different medicinal plants and metabolites have different responses to soil pH. For example, in the range of soil pH 4.5 ~ 9.5, the content of active ingredients in *P.multiflorum* tubers decreased with the increase of pH, and the contents of stilbene glycoside and bound anthraquinone in *P.multiflorum* tubers reached the highest at pH 4.5 (Leng et al., 2020). Zou found that the content of total flavonoids, oleanolic acid and ursolic acid in *G. longituba* was highest at pH 6.5, while the content of rosmarinic acid was highest at pH 7.5 (Zou et al., 2019). In this study, a variety of flavonoids, glycosides, alkaloids and organic acids were significantly correlated with soil total salt content and pH such as silybin (M453T51_1), catalposide (M465T412), poncirin (M617T328_2), plantamajoside (M639T418), isorhamnetin 3-galactoside (M501T353), harmane (M181T170), phenylalanine betaine (M208T197), chicoric acid (M497T336) etc. These secondary metabolites may be important substances for SDL to respond to changes in soil pH and salt content. In conclusion, soil physicochemical properties may be the most important habitat factor affecting SDL metabolites.

Annual precipitation and temperature are also important habitat factors that are highly correlated with various pivot metabolites in this study. Precipitation can reflect the water environment of plant growth. Water is an important medium linking the atmosphere, the soil to the plant and the metabolic activities of the plant, which affects plant physiological and biochemical processes such as photosynthesis, respiration, oxidation and secondary metabolic activities in plants (Bohnert et al., 1995; Jat and Gajbhiye, 2017). Lang found that the root growth of SDL was inhibited to a certain extent during moderate to severe drought stress (40% field water holding capacity), which affected the yield; however, drought stress significantly increased the content of total flavonoids, total saponins and other secondary metabolites in SDL herbs (Lang et al., 2014). This study found that flavonoids deoxyrhapontin (M449T356) and saponins astragaloside ii (M871T33_3) showed a significant correlation with annual precipitation, which further verifying the previous results. Temperature can directly affect the growth and development, physiological activity, harvest time and the accumulation of compositions of medicinal plants. In particular, during critical growth periods, it can alter the activity of relevant enzymes in the medicinal plant, which directly affects the level of secondary metabolism of plants (Eguchi et al., 2019). Furthermore, elevation and latitude can indirectly affect the growth and development of medicinal plants by influencing habitat factors such as temperature and precipitation (Joshi et al., 2021). In summary, the significant differences of SDL metabolites from different origins may be due to the combined and complex effects of habitat factors such as soil physical and chemical properties, annual precipitation, annual mean temperature, altitude and latitude. However, what kind of habitat is conducive to the growth of SDL and the formation of medicinal quality, and how to promote the high-quality production of SDL through scientific cultivation measures, need deeper thinking and research.

“Simulative Habitat Cultivation” is the core model of ecological cultivation of Chinese herbs proposed by academician Luqi Huang and researcher Lanping Guo. Based on the long-term adaptation of medicinal plants to specific environmental stresses, it simulated various environmental factors of wild medicinal plants, especially the original habitat of genuine herbs, and then balance the growth and development of Chinese herbs and secondary metabolism by utilizing scientific design and clever human intervention, thus achieving the optimal layout and high-quality development of genuine Chinese herbs. Especially in the absence of more research bases and special production purposes, “Simulative Habitat Cultivation” of genuine medicinal materials can be used as a basic model for high-yield and high-quality production of Chinese herbs (Guo L. et al., 2020). SDL has a long history and widely application. However, in modern research, basic research on SDL is lagging behind or in the blank, such as the research on efficacy mechanisms, material basis, quality markers and habitat stress response mechanisms, etc. But, in recent modern research, the basic research on its mechanism of efficacy, therapeutic material basis, quality markers and the response mechanism under environmental stress of SDL is at a preliminary stage. This has led to a lack of effective evaluation indicators in the production and quality evaluation of SDL, which in turn has hindered the industrial development and resource utilisation of SDL. Therefore, in the absence of a research base, the “simulated biotope” model combined with metabolomics technology offers a new idea for the more scientific production of SDL.

During the period from 2017 to 2022, the author’s research group conducted several surveys on SDL resources in and around Ningxia (Li et al., 2022). The survey found that wild SDL was concentrated in Lingwu City and Yanchi County in Ningxia and Etuokeqianqi County in Inner Mongolia, which all belong to the wind-sand arid area and have similar habitat characteristics such as soil physicochemical properties, climate and spatial distribution. Metabolites of SDL collected in these regions also have relatively similar characteristics. And the content of various active substances in SDL collected from Lingwu City (LW) was significantly higher than that in SDLcollected from cultivated origin (TX), such as beta.-sitosterol (M397T42), trigonelline (M138T291_2), betaine (M118T277_2), fustin (M269T36), rotenone (M241T189), arctiin (M557T165) and loganic acid (M399T284_2). Therefore, the wind-sand arid area can be used as the preferred ecological area for SDL “Simulative Habitat Cultivation” production. In addition, the SDL of LW, YC and ETKQQ are all distributed in the desert grassland in the region. So the environmental characteristics of the desert grassland in the arid area may be the main habitat characteristics in the formation of SDL genuineness, such as less rain, alkaline sand soil, etc.

The collection site of DWK is mainly located near the Shitanjing coal mine, which is another area of concentrated distribution of wild SDL. The results revealed that the habitat characteristics of this collection site, especially the physicochemical properties of the soil, were significantly different from those of the other wild distribution areas, and the characteristics of metabolites were also significantly different from those of the others. At the meanwhile, the survey also showed that SDL is the dominant species in these regions, which is adapted to grow in the specific environment. A large number of studies have confirmed that plants growing in a specific environment for a long time are subject to the combined influence of various ecological factors in the environment, which may cause changes in genetic material such as mutations in plant DNA and aberrations in chromosome structure and number, and then change metabolic regulatory enzymes, resulting in variation in the products of secondary metabolism (Cao et al., 2021; Zhang et al., 2021). In the present study, it is worthwhile to pay attention to and study in more depth whether the distinct habitats of DWK have affected the genetic material of SDL, thus causing metabolites of SDL to be significantly different from other samples, or whether new varieties have been produced.

As the largest concentrated cultivation area of SDL, Tongxin County has a long history of cultivation and a good production and processing base, and was awarded the “Tongxin Yinchai hu” geographical indication certification for agricultural products in 2018, which is recognized as the genuine origin of cultivated SDL. This research found some differences in characteristics of metabolites between SDL in Tongxin County and samples collected in the wild. However, key habitat indicators such as soil total salt content, pH value, annual precipitation and average annual temperature of the local natural habitat were very close to those of the wild SDL habitat, with only some differences in soil texture, which may be the main reason for the differences between TX and wild SDL. The soil of the TX habitat is clayey soil, rather than the sandy soil of the wild habitat. Therefore, it is recommended that during the production and planting of SDL in Tongxin County, measures such as deep ploughing and applying soil cavitation amendments to increase soil porosity, so as to achieve the purpose of “Simulative Habitat Cultivation” and guarantee better quality of SDL.

## 5 Conclusion

In this study, a total of 1586 metabolites were identified in SDLs from nine habitat by the UHPLC-Q-TOF MS based metabolomics. Differential metabolites among nine origins were analyzed through multivariate statistics and the correlations between metabolites and the habitat factors were also investigated and discussed. The results showed that SDLs from different habitats had various metabolites, and the samples with similar habitat factors also showed similar metabolite characteristics. These differential metabolites are mainly some lipids and lipid molecules, organic acids and their derivatives, phenylpropane and polyketone compounds, etc. Further more, 1586 metabolites were clustered into seven co-expression modules by the CNA. And the correlation analysis of seven modules with thirteen habitat factors showed that three metabolite modules(MEturquoise, MEbrown and MEblue) showed significant correlations with different habitat factors and 104 species hub metabolites were further screened out. Soil texture, soil pH value and soil total salt content were selected as the most dominant habitat factors affecting SDL metabolites, and then followed by annual precipitation and temperature, elevation and latitude. The research provides theoretical and practical significance for guiding the construction of genuine producing areas, the scientific production and “Simulative Habitat Cultivation” for SDL.

## Data availability statement

The original contributions presented in the study are included in the article/**Supplementary Material**. Further inquiries can be directed to the corresponding authors.

## Author contributions

The manuscript was written through contributions of all authors. ZKL, resource survey, Sample collection, Methodology, Sample detection, Writing-review and editing, Data analysis, Visualization; HW and LS, Resource survey, sample collection, sample detection; LF and HSL, writing-review and editing; HYL, Meteorological data collection; YPL, Metabonomic analysis; YQL, sample testing, data analysis; YY and GGT, visualization; XGM, conceived, revised and supervised the manuscript; LP, conceived and designed the study, methodology, supervision, funding acquisition. All authors contributed to the article and approved the submitted version.

## Funding

This study was supported by the Key Research and Development Program of Ningxia (No.2021BEG02042) and Ningxia Natural Science Foundation (No.2021AAC03103).

## Acknowledgments

The authors acknowledge Shanghai Applied Protein Technology Co., Ltd. for their support for metabolite testing, and Ma Li (School of Foreign Chinese, Ningxia University) for her help in language translation.

## Conflict of interest

The authors declare that the research was conducted in the absence of any commercial or financial relationships that could be construed as a potential conflict of interest.

## Publisher’s note

All claims expressed in this article are solely those of the authors and do not necessarily represent those of their affiliated organizations, or those of the publisher, the editors and the reviewers. Any product that may be evaluated in this article, or claim that may be made by its manufacturer, is not guaranteed or endorsed by the publisher.

## Supplementary material

Additional data relevant to this paper can be found in the Annexes.

Supplementary Figure 1 Quality control analysis of metabolite detection. (**A** is TIC diagram in neg ion mode; **B** is characteristic peak variation coefficient in neg ion mode; **C** is the PCA of metabolites detected in neg ion mode.).

Supplementary Figure 2 Volcano plot analysis of different comparison groups (**A, C, E, G** were TX vs PY, TX vs HSP, TX vs DWK, and TX vs ALSZQ in pos mode, respectively. **B, D, F, H** were TX vs PY, TX vs HSP, TX vs DWK, and TX vs ALSZQ in pos mode, respectively.).

Supplementary Figure 3 OPLS-DA and permutation test (pos). (**A, C, E, G** are OPLS-DA of TX vs PY, TX vs HSP, TX vs DWK, and TX vs ALSZQ, respectively; **B, D, F, H** are permutation test of TX vs PY, TX vs HSP, TX vs DWK, and TX vs ALSZQ, respectively.).

Supplementary Figure 4 OPLS-DA and permutation test (neg). (**A, C, E, G** are OPLS-DA of TX vs PY, TX vs HSP, TX vs DWK, and TX vs ALSZQ, respectively; **B, D, F, H** are permutation test of TX vs PY, TX vs HSP, TX vs DWK, and TX vs ALSZQ, respectively.).

Supplementary Figure 5 FC analysis histogram of SCMs in different comparison groups (**A, C, E, G** are FC analysis of TX vs PY, TX vs HSP, TX vs DWK, and TX vs ALSZQ in pos mode, respectively; **B, D, F, H** are FC analysis of TX vs PY, TX vs HSP, TX vs DWK, and TX vs ALSZQ in neg mode, respectively.).
