# Supplementary figures and images for: Comparative metabolomics provides novel insights into correlation between dominant habitat factors and constituents of Stellaria Radix (Stellaria dichotoma L. var. lanceolata Bge.)
Source: Front Plant Sci. 2022 Nov 25;13:1035712. doi: 10.3389/fpls.2022.1035712 (PMC9733579; doi:10.3389/fpls.2022.1035712)

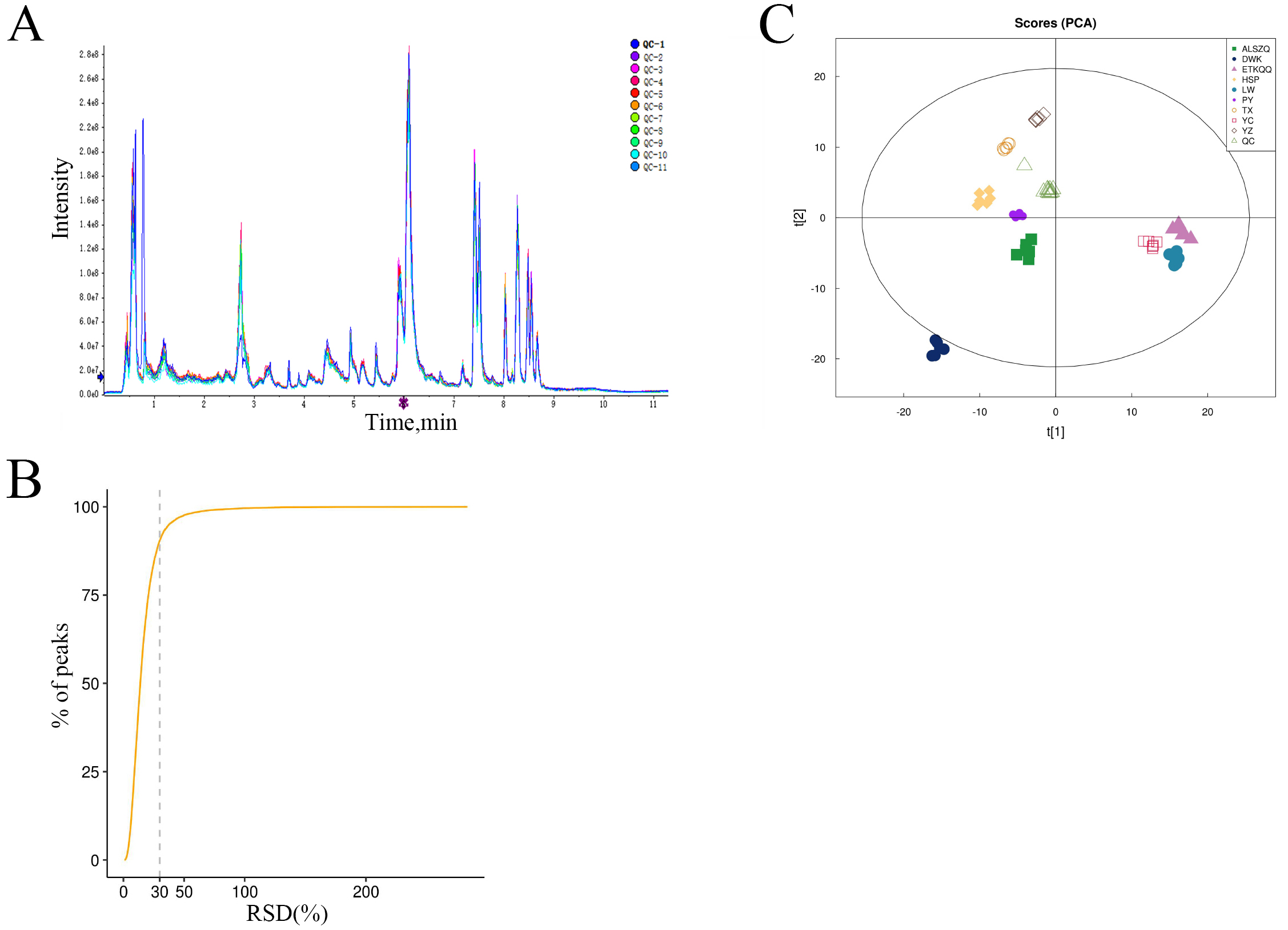

Supplement: Supplementary Figure 1 — Quality control analysis of metabolite detection. (A is TIC diagram in neg ion mode; B is characteristic peak variation coefficient in neg ion mode; C is the PCA of metabolites detected in neg ion mode.). [file Image_1.jpeg]

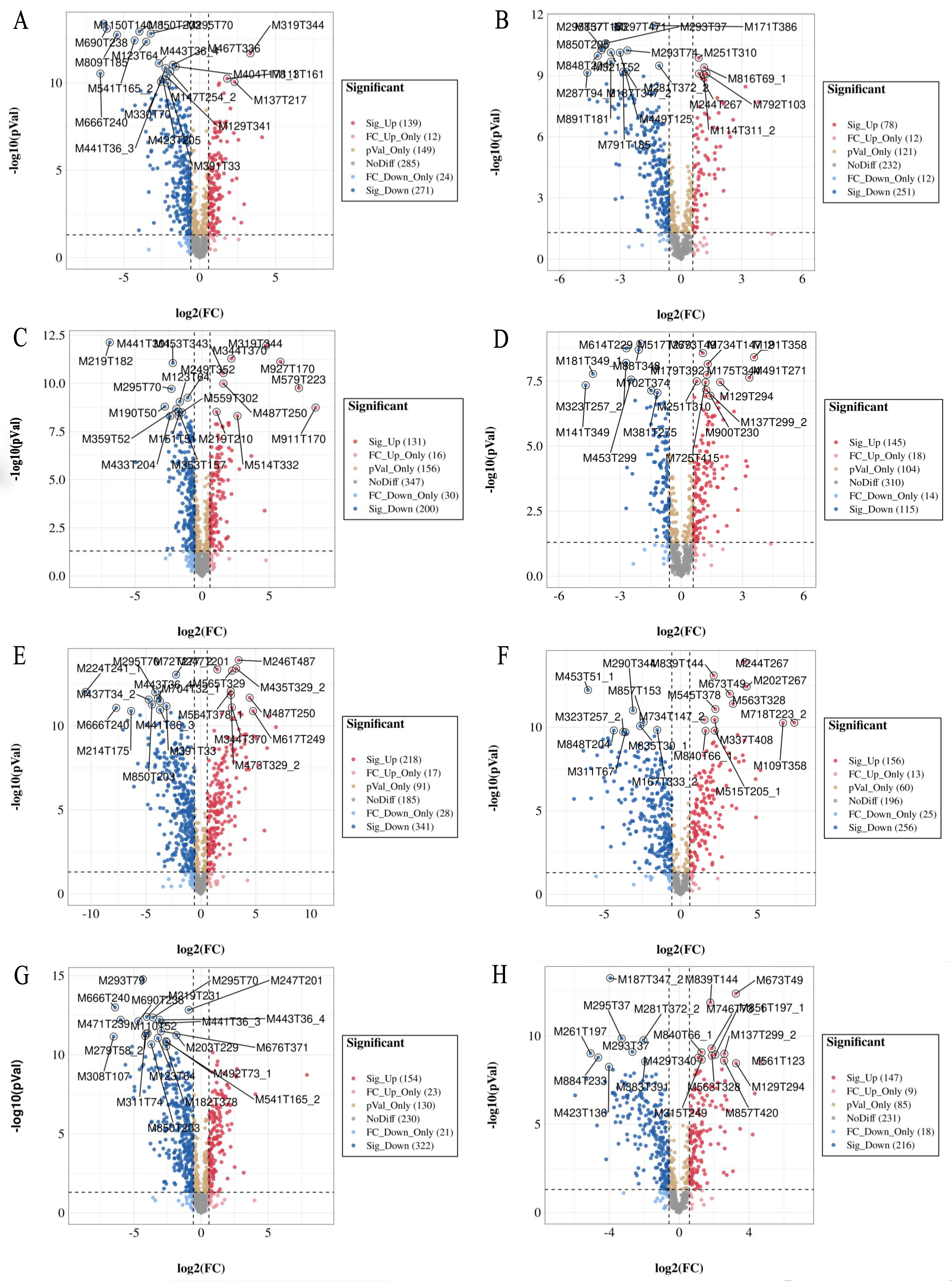

Supplement: Supplementary Figure 2 — Volcano plot analysis of different comparison groups (A, C, E, G were TX vs PY, TX vs HSP, TX vs DWK, and TX vs ALSZQ in pos mode, respectively. B, D, F, H were TX vs PY, TX vs HSP, TX vs DWK, and TX vs ALSZQ in pos mode, respectively.). [file Image_2.png]

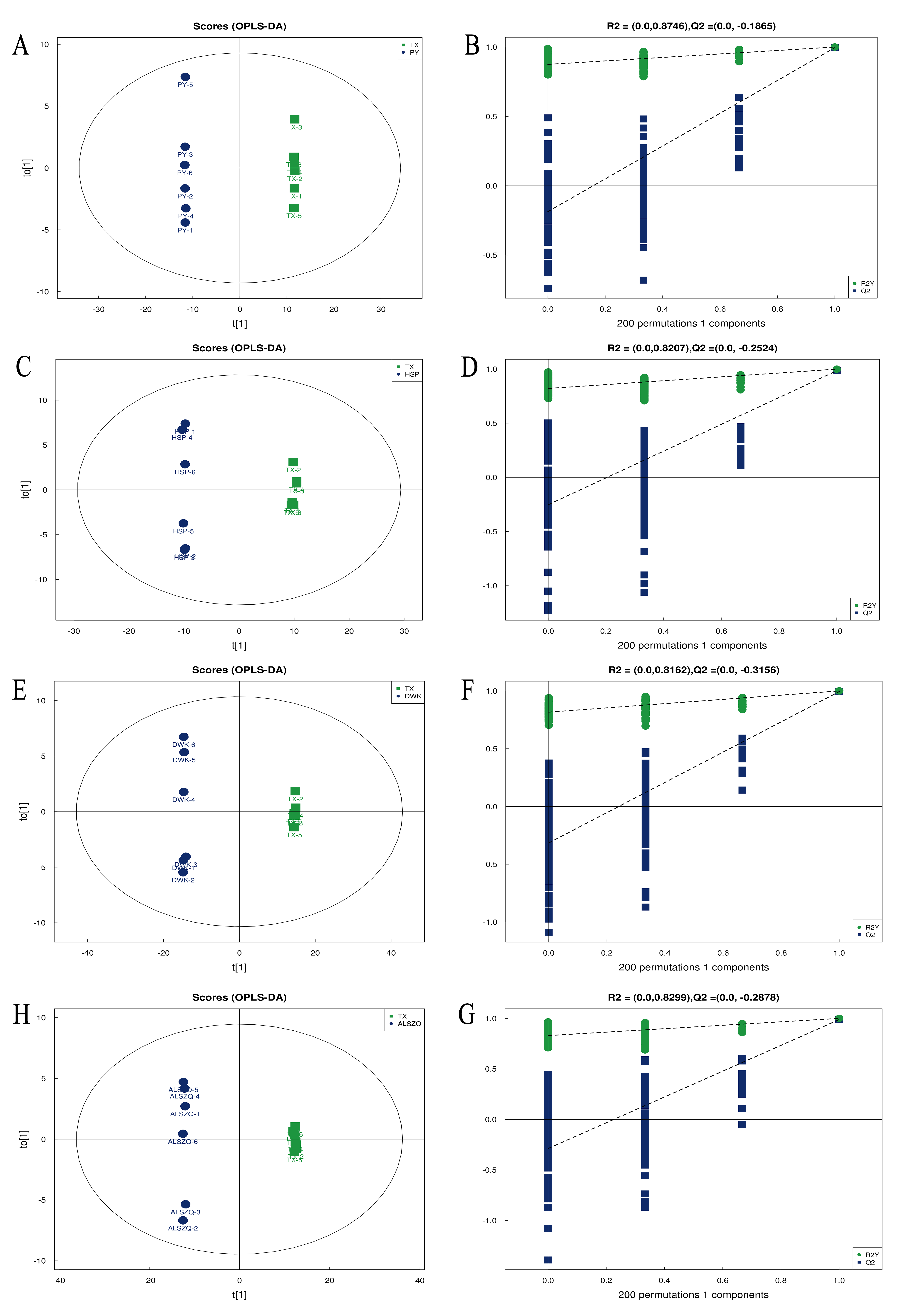

Supplement: Supplementary Figure 3 — OPLS-DA and permutation test (pos). (A, C, E, G are OPLS-DA of TX vs PY, TX vs HSP, TX vs DWK, and TX vs ALSZQ, respectively; B, D, F, H are permutation test of TX vs PY, TX vs HSP, TX vs DWK, and TX vs ALSZQ, respectively.). [file Image_3.png]

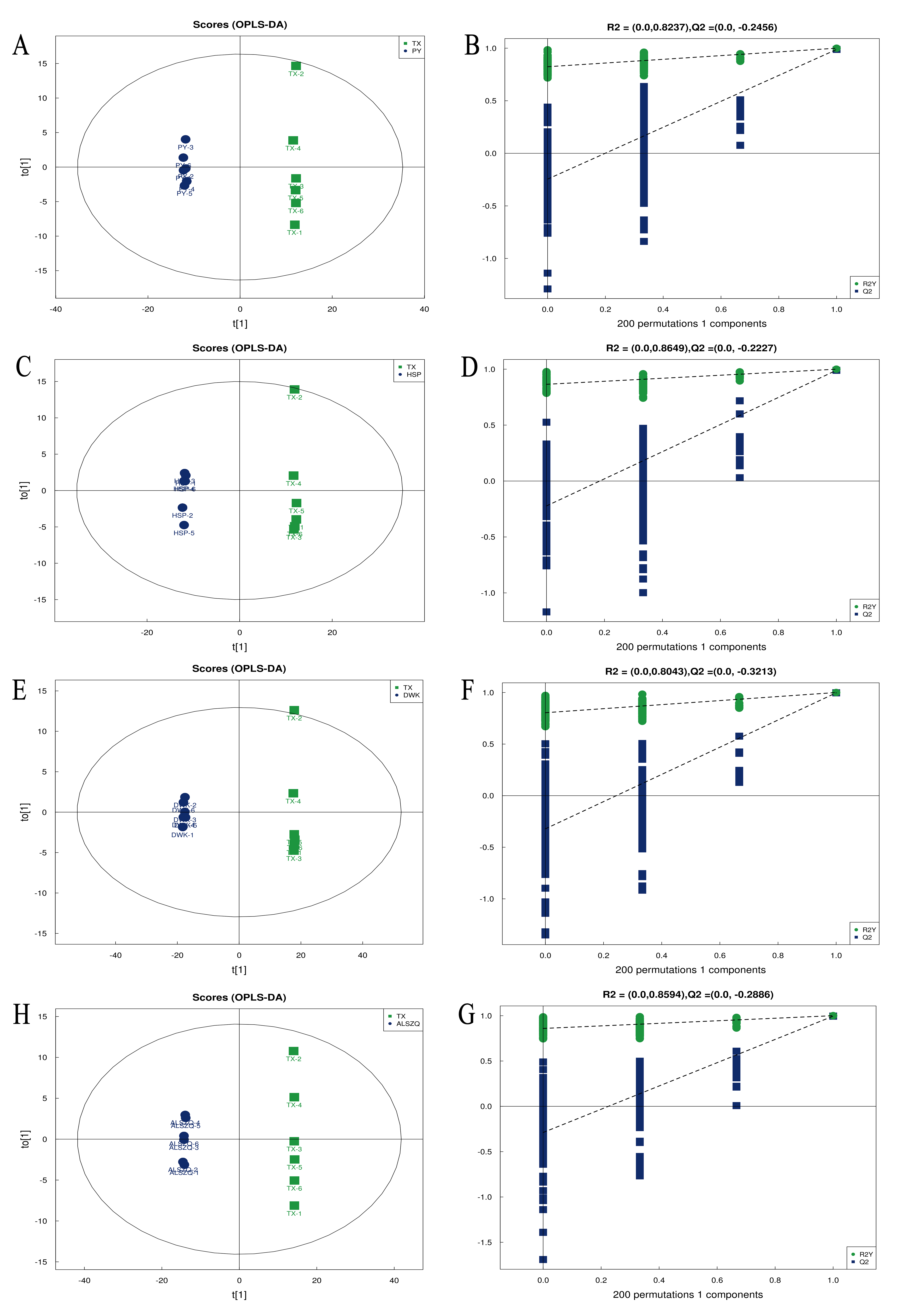

Supplement: Supplementary Figure 4 — OPLS-DA and permutation test (neg). (A, C, E, G are OPLS-DA of TX vs PY, TX vs HSP, TX vs DWK, and TX vs ALSZQ, respectively; B, D, F, H are permutation test of TX vs PY, TX vs HSP, TX vs DWK, and TX vs ALSZQ, respectively.). [file Image_4.png]

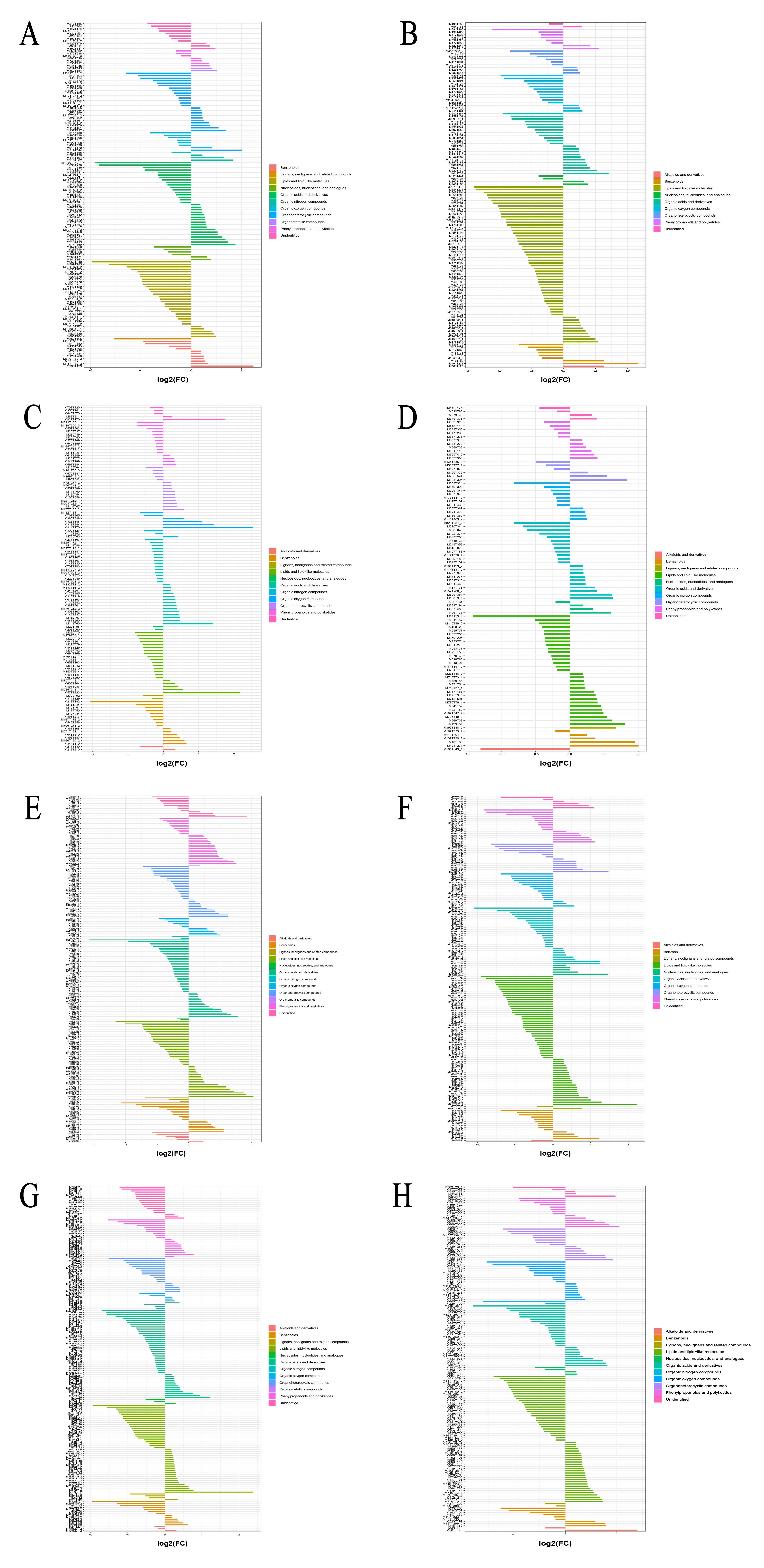

Supplement: Supplementary Figure 5 — FC analysis histogram of SCMs in different comparison groups (A, C, E, G are FC analysis of TX vs PY, TX vs HSP, TX vs DWK, and TX vs ALSZQ in pos mode, respectively; B, D, F, H are FC analysis of TX vs PY, TX vs HSP, TX vs DWK, and TX vs ALSZQ in neg mode, respectively.). [file Image_5.png]
